# Surveillance of Nipah virus in *Pteropus medius* of Kerala state, India, 2023

**DOI:** 10.3389/fmicb.2024.1342170

**Published:** 2024-03-05

**Authors:** R. Balasubramanian, Sreelekshmy Mohandas, Ullas P. Thankappan, Anita Shete, Dilip Patil, Kannan Sabarinath, Basavaraj Mathapati, Rima Sahay, Deepak Patil, Pragya D. Yadav

**Affiliations:** ^1^Field Unit, ICMR-National Institute of Virology, Alappuzha, India; ^2^Maximum Containment Facility, ICMR-National Institute of Virology, Pune, India; ^3^Encephalitis Group, ICMR-National Institute of Virology, Pune, India; ^4^Animal House, ICMR-National Institute of Virology, Pune, India; ^5^Field Unit, ICMR-National Institute of Virology, Dibrugarh, India

**Keywords:** Nipah virus, *Pteropus medius*, India, Kerala, seroprevalence

## Abstract

**Introduction:**

Since 2018, the Indian state of Kerala has reported four Nipah virus (NiV) disease outbreaks, raising concerns about NiV spillover from bats to the human population. Considering this, a cross-sectional study was undertaken in the *Pteropus medius* bat population around the Nipah virus-affected regions of Kozhikode, Kerala, India, during February, July, and September 2023.

**Methods:**

Throat swabs, rectal swabs, and organ samples were collected from bats to test for NiV using the real-time reverse transcriptase polymerase chain reaction (RT-PCR), while serum samples were screened for anti-Nipah IgG antibodies through ELISA.

**Results:**

An overall seroprevalence of 20.9% was observed in 272 *P. medius* bats tested. The throat and rectal swab samples of 321 bats were negative for NiV RNA. However, 4 of 44 *P. medius* bats tested positive for NiV in their liver/spleen samples. The partial N gene retrieved showed more than 99% similarity with the earlier reported NiV genome from Kerala state, India.

**Discussion:**

The findings of the study caution that there is a spillover risk in the region and necessary precautions should be taken.

## 1 Introduction

Nipah virus (NiV) disease is a highly fatal zoonotic viral infection caused by a paramyxovirus. NiV disease outbreaks have been reported in Malaysia, Singapore, Bangladesh, the Philippines, and India (Ang et al., 2018). Frugivorous bats of the *Pteropus* genus are considered the reservoir for NiV (Epstein et al., 2020). In Malaysia and Singapore, transmission has occurred via spillover from fruit bats to the pig populations and then to humans (Chua et al., 1999; Paton et al., 1999). In Bangladesh, the consumption of date palm sap contaminated with bat secretions is considered to be the route of transmission (Montgomery et al., 2008). India has experienced six outbreaks in the past, namely, two in the eastern state of West Bengal bordering the Nipah belt of Bangladesh and four from the Southern state of Kerala (World Health Organization, 2023). The surveillance of *Pteropus medius* bats in India has revealed the presence of NiV from the outbreak-reported regions, although the route of transmission remains obscure (Yadav et al., 2018, 2019; Sudeep et al., 2021).

The first outbreak of NiV in India was reported in 2001 from Siliguri, West Bengal, with a case fatality rate (CFR) of 74%. Another outbreak occurred in the Nadia district of the same state in 2007 with 100% CFR (Chadha et al., 2006; Arankalle et al., 2011). Subsequently, the ICMR-National Institute of Virology (NIV), Pune, conducted surveillance in fruit bats of the West Bengal and adjoining states of the Northeast region of India. In 2010, *P. medius* from Maynaguri, West Bengal, showed the presence of NiV (Yadav et al., 2012). Subsequently in 2015, the presence of NiV was detected among *P. medius* bats collected from Cooch Bihar district, West Bengal, and Dhubri district, Assam (Yadav et al., 2018). The other four outbreaks occurred in Kerala, which is in the southernmost part of the country, in recent years (Yadav et al., 2019, 2022; Sudeep et al., 2021; World Health Organization, 2023). During the NiV outbreak in 2018, 23 laboratory-confirmed cases and 21 deaths were reported. The surveillance of *P. medius* bats in the vicinity of the index case house revealed 25% (13/52 bats) positivity for NiV RNA in the throat and rectal swab samples collected. Although the direct link of transmission from bats to humans could not be established, the viral genome obtained from bats showed a close homology with the NiV sequences obtained from patients in Kerala (Yadav et al., 2019). The phylogenetic analysis showed the NiV Indian sequences forming a separate subcluster within the Bangladesh genotype.

In 2019, a single case of NiV infection was reported in the Ernakulam district of Kerala, in which the patient survived. The source of the infection could not be determined, but screening fruit bats in the outbreak area revealed the presence of NiV in the rectal swab and organ samples (Sudeep et al., 2021). Prompt interventions and preventive actions were taken in time to curtail the further spread of infection during the outbreak. The Kozhikode district of Kerala witnessed a second outbreak in September 2021, with a single case reported. The *P. medius* sampled from the area during the outbreak showed NiV seropositivity (Yadav et al., 2022). The presence of antibodies against NiV has been demonstrated in *P. medius* bats sampled from other southern states of India, indicating a risk of spillover in the region (Gokhale et al., 2022). In September 2023, another outbreak occurred in the Kozhikode district, with a total of six cases and two deaths (World Health Organization, 2023). The source of infection for the index case remains unknown as in the previous outbreaks.

The state of Kerala has experienced four outbreaks in the recent past, with outbreaks tending to cluster between the months of May and September (Yadav et al., 2019; Sudeep et al., 2021; Yadav et al., 2022; World Health Organization, 2023). This pattern of outbreak occurrences has led to speculations about the seasonality of virus shedding in bats. To understand the prevalence of the NiV in *P. medius*, we conducted a repeated cross-sectional study in and around Kozhikode district in Kerala where multiple outbreaks have occurred.

## 2 Materials and methods

### 2.1 Sample collection

**Figure 1:**
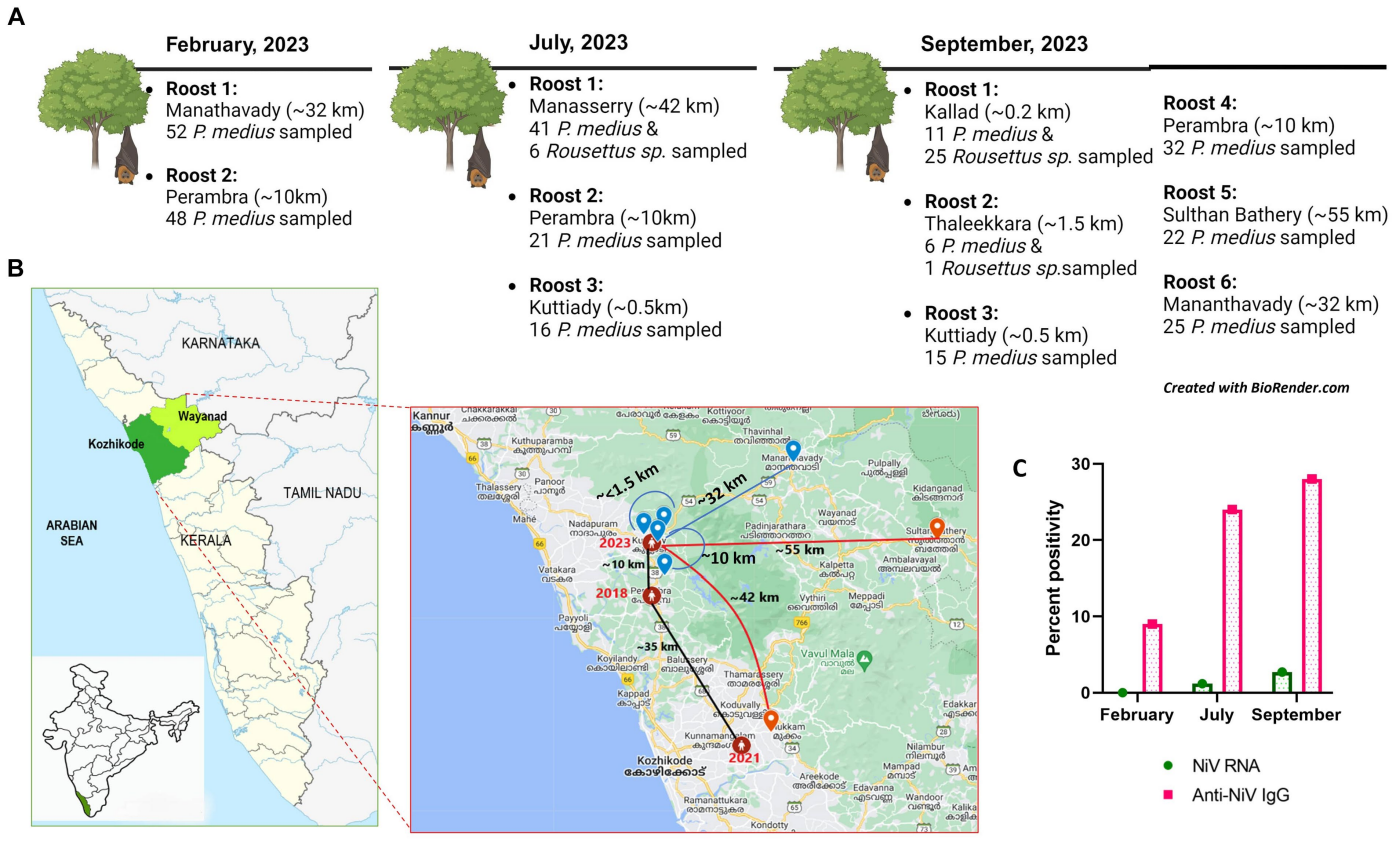
**(A)** The bat sampling sites in Kerala with details of the location of the roost (aerial distance from the Nipah virus index case house of 2023) and the number of bats sampled in February, July, and September of 2023. **(B)** The geographical map of Kerala with the Kozhikode (green) and Wayanad (light green) districts from which sample collection was performed is highlighted, and in the inset, the locations of Nipah virus outbreak reported areas (of the years 2018, 2019, and 2021 marked in red connected with black line showing the distance among each other) and the bat sampling roost locations (blue icon and red icon) with the aerial distance plotted from the 2023 Nipah virus outbreak location as blue lines (to roosts which were Nipah virus negative) and red lines (to roosts which showed Nipah viral RNA positivity). **(C)** The graphical representation of NiV RNA and antibody percent positivity estimated during the months of February, July, and September from the outbreak region in 2023.

With the requisite permissions from the institutional ethical committee and state forest authorities, the study was initiated. *P. medius* roosting sites around the NiV outbreak areas in Kozhikode were identified. The sampling was performed during February, July, and September 2023. The collection during September was conducted as part of the NiV outbreak investigation in the Kozhikode district. The bats were trapped using mist nets and were anesthetized before sample collection (throat swab, rectal swab, and serum) as described earlier (Sudeep et al., 2021). The swab samples were collected using thin nylon flocked swabs in 1 mL of viral transport media (HiMedia, Mumbai, India). The blood samples were collected from the cephalic vein using a 25 G needle attached to a 1-ml syringe. A total of 289 *P. medius* bats and 32 *Rousettus* sp. bats were sampled during the study in February (*P. medius*, *n* = 100), July (*P. medius*, *n* = 78, *Rousettus* sp., *n* = 6), and September (*P. medius*, *n* = 111, *Rousettus* sp., =26) from the locations described in Figures 1A,B. The bats were released to their natural habitat after anesthesia recovery. During each collection time point, i.e., 14 *P. medius* in February, 6 *P. medius* in July, and 24 *P. medius* in September were euthanized and transported to the containment laboratory for organ collection. The bat species were identified by their morphology and mitochondrial cytochrome b gene PCR (Yadav et al., 2022).

### 2.2 Nipah virus real-time RT-PCR

The throat swab (*n* = 321), rectal swab (*n* = 321), and organ samples (liver, spleen, kidney, lungs, heart, and intestines of 44 bats) were screened for NiV RNA using the N gene-specific real-time RT-PCR (Guillaume et al., 2004). The organ samples collected from bats in the containment facility were weighed and homogenized using a tissue homogenizer (Qiagen, Germany). The RNA was extracted from the swabs and organ homogenates using the MagMAX Viral RNA Isolation Kit (Thermo Scientific, USA) as per the manufacturer’s instructions. TaqMan chemistry-based real-time RT-PCR was carried out with published NiV-specific primers and probes targeting the N gene. The results were recorded as the cycle threshold (CT) values. The assay cutoff was determined as described by Guillaume et al. (2004). Any sample with a CT value of <38.0 was considered positive.

### 2.3 Sanger sequencing

Sequencing was performed for positive samples using the Sanger chain-termination method. The total nucleic acid was extracted from specimens using the QIAamp Viral RNA Mini Kit (Qiagen, Germany) column purification; 5 mL of the RNA eluted from each positive specimen was used to set up the conventional nested RT-PCR with the in-house designed primers specific for the N gene. For the first PCR of product size 605 base pairs (bp), forward: CGTGGTTATCTTGAACCTATGTACTTCAG and reverse: CGCAACTTTAATGTAATTGGTCCCTTAGTG were used, and for the second PCR of 342 bp, forward: CAGAGAAGCTAAATTTGCTGCAGGAGG and reverse: TCACACATCAGCTCTGACAAAGTCAAG were used. The PCR products were electrophoresed on 1.5% agarose gel, and bands of size 342 bp were excised and further purified through the QIAquick Gel Extraction Kit (Qiagen, Germany). Cyclic Sequencing PCR using the Big Dye™ Terminator Cycle Sequencing Kit (Invitrogen) was used to set up the sequencing PCRs on purified cDNA products. Using the DyeEx 2.0 Kit (Qiagen), the sequencing PCR products were purified and sequenced using the ABI PRISM® 3,100 Automated DNA Sequencer (Applied Biosystems, USA) platform. Sequencher 5.1 software (Accelrys Inc., USA) was used to analyze the chromatogram data obtained. The sequences were submitted to GenBank.

### 2.4 Phylogenetic analysis

A phylogenetic analysis was conducted using a subset of Nipah virus sequences (obtained from humans, bats, and pigs) available on GenBank from Malaysia, Bangladesh, India, Cambodia, and Thailand along with the partial N gene sequence obtained through Sanger sequencing from the bat samples of the present study. The reference sequences of the Nipah virus (NC_002728.1) from the National Center for Biotechnology Information (NCBI) database along with 22 NiV sequences from previous outbreaks of India (2007, 2018, 2019, 2021, and 2023), 10 representative sequences from Bangladesh, 1 sequence from Cambodia, 1 from Thailand, and 9 representative sequences from Malaysia were used for the phylogenetic analysis. The alignment of multiple genomes was conducted using the MAFFT tool, and MEGAX was employed for the visualization of the alignment (Katoh et al., 2019). To ensure proper alignment, all sequences were truncated to an equal length of 342 nucleotides. Subsequently, the construction of the phylogenetic tree was carried out utilizing the maximumlikelihood method using the Tamura and Nei, 1993 (TN) + Empirical base frequencies (F) model implemented in IQTREE, incorporating 1,000 ultrafast bootstrap replicates (Minh et al., 2020). The resulting phylogenetic tree was visualized using iTOL (Interactive Tree of Life) (Letunic and Bork, 2021).

### 2.5 Indirect anti-Nipah bat IgG ELISA

The serum samples of *P. medius* (*n* = 272) and *Rousettus* sp. (*n* = 10) were tested for antibodies using anti-Nipah bat IgG ELISA. Antibodies against NiV from bat sera samples were detected using an in-house developed and validated indirect enzyme-linked immunosorbent assay (Gokhale et al., 2022). The sera samples were heat-inactivated and used for the assay. The plate coated with NiV antigen was incubated overnight at 4°C. The plates were washed thrice, and the serum samples were added and incubated at 37°C for 60 min. The plates were washed four times, and anti-bat IgG HRP conjugate was added and incubated for 60 min. Then, 3,3′,5,5′-Tetramethylbenzidine substrate was added to each plate and incubated at 37°C for 30 min. The reaction was stopped with 1 N H_2_SO_4_, and the plate was read at 450 nm in an ELISA reader. The OD value greater than or equal to 0.2 and the positive/negative ratio more than or equal to 3.0 were considered positive for the presence of anti-NiV IgG antibodies.

### 2.6 Virus neutralization

A subset of bat serum samples (~50% of total seropositive samples; *n* = 23 and 12 negative samples) was confirmed for virus neutralization in Vero-CCL-81 cell monolayers in 96-well plates. The bat serum samples were heat-inactivated at 56°C for 60 min. Two-fold dilutions of the bat sera were made using the sterile tissue culture media. Nipah virus (GenBank accession ID: MH523642) of 100 TCID50 dilution was added to the diluted serum samples, and the virus–serum mixture was incubated at 37°C for 1 h in a CO_2_ incubator. After incubation, 100 mL of the virus–serum mixture was added to the Vero cell monolayer in 96-well plates. Tissue culture media were added to the cell control wells. As positive and negative controls, anti-Nipah IgG positive and negative mice sera were used, respectively. The plates were incubated in a CO_2_ incubator for 5 days, and the neutralization titer was calculated by observation of cytopathic changes. The assay was performed in duplicate for each sample.

### 2.7 Virus isolation

Nipah virus isolation was attempted from samples positive for NiV RNA using Vero cells (ATCC® CCL-81™, ATCC, USA) and laboratory mice in the BSL-4 facility. For the virus isolation in Vero (ATCC® CCL-81™) cells, the positive samples were inoculated to 24-well plates (with 70–80% cell confluency) and were incubated for 1 h. The plates were washed with PBS after 1 h, and fresh media with 2% fetal bovine serum were added. The plates were incubated in a CO_2_ incubator for 5 days and were observed for cytopathic effects every day. The samples were blindly passaged two times in Vero (ATCC® CCL-81™) cells. After each passage, the cell culture fluid was tested for NiV using real-time RT-PCR. For the virus isolation in mice, 3- to 4-day-old CD1 mice procured from the laboratory animal facility, ICMR-NIV, Pune, were used. The intracranial inoculation of positive samples was performed, and the mice were observed for sickness for 7 days. After 7 days, the brains of mice were collected and homogenized in sterile media and processed for NiV real-time RT-PCR.

## 3 Results

### 3.1 Nipah viral RNA detection in *Pteropus medius*

**Figure 2:**
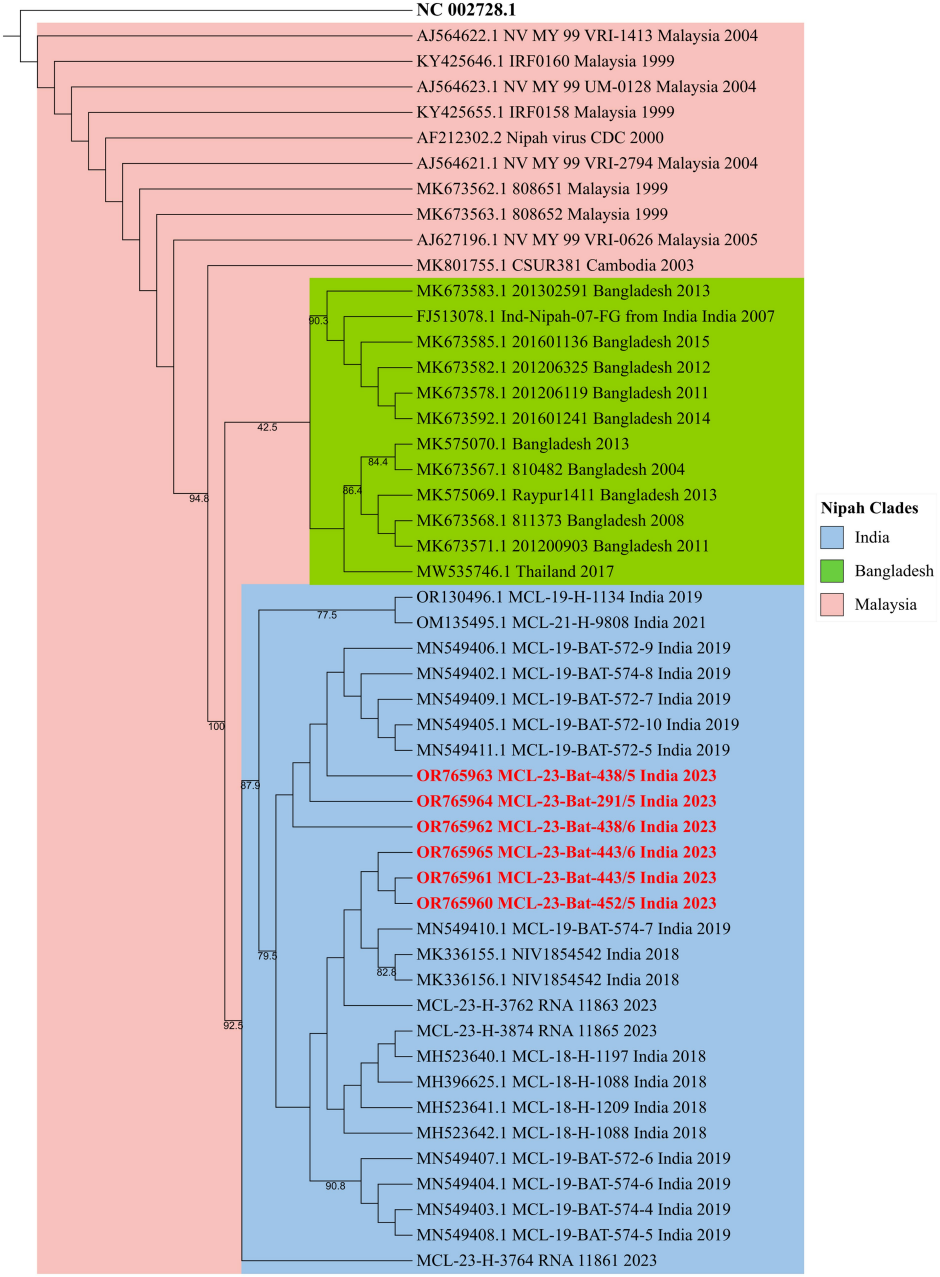
The phylogenetic tree of the partial N gene sequences of 342 nucleotide length generated by the maximum-likelihood method using the TN + F model in IQTREE. The Nipah virus sequences retrieved (highlighted in red) from the *P. medius* from Kerala 2023 show clustering with the NiV-Indian genotype sequences.

All the throat and rectal swab samples collected from *P. medius* bats (*n* = 289) during different months were negative for NiV RNA. The spleen sample of one *P. medius* collected during July 2023 from Manassery (~42 km from the 2023 outbreak index case location) and the spleen/kidney samples collected from three *P. medius* in September 2023 from Sulthan Bathery (~55 km from the index case location) were found positive for NiV RNA (Table 1). The partial N gene retrieved from these samples (GenBank accession no.: OR765960, OR765961, OR765962, OR765963, OR765964, and OR765965) showed more than 99% similarity to the earlier reported NiV sequences (from bats and humans) from Kerala belonging to the cluster of NiV Indian sequences (Figure 2). The virus isolation attempts in Vero (ATCC® CCL-81™) cells and mice model were not successful. All the *Rousettus* sp. (*n* = 32) bat samples were found negative for NiV RNA.

**Table 1:** The sample collection details and the test results of NiV real-time RT-PCR and anti-NiV IgG ELISA during the study.

Sl no.	Month and year of collection	Sample collection site (Place, District)	*Pteropus medius*			Nipah Real-Time RT PCR (Positive/total tested)				IgG ELISA (Positive/total tested)
			Number sampled	Male/Female (Number)	Body weight in grams (Mean ± SD)	Throat Swab	Rectal Swab	Spleen	Kidney	
1	February, 2023	Mananthavady, Wayanad	52	M (28) *F* (24)	552 ± 160 580 ± 119	0/52	0/52	0/4	0/4	07/46
		Perambra, Kozhikode	48	M (30) *F* (18)	559 ± 99 499 ± 93	0/48	0/48	0/10	0/10	01/42
2	July, 2023	Manassery, Kozhikode	41	M (21) *F* (20)	716 ± 239 513 ± 148	0/41	0/41	1/3	0/3	16/39
		Perambra, Kozhikode	21	M (13) *F* (8)	618 ± 212 504 ± 114	0/21	0/21	0/1	0/1	02/19
		Kuttiady, Kozhikode	16	M (10) *F* (6)	690 ± 205 405 ± 119	0/16	0/16	0/2	0/2	0/16
3	September, 2023	Kallad, Kozhikode	11	M (4) *F* (7)	528 ± 124 442 ± 108	0/11	0/11	0/1	0/1	1/11
		Thaleekkara, Kozhikode	6	M (2) *F* (4)	638 ± 120 619 ± 103	0/6	0/6	-	-	2/6
		Kuttiady, Kozhikode	15	M (9) F (6)	578 ± 124 430 ± 155	0/15	0/15	0/2	0/2	1/15
		Perambra, Kozhikode	32	M (20) *F* (12)	630 ± 140 555 ± 139	0/32	0/32	-	-	14/31
		Sulthan Bathery, Wayanad	22	M (13) *F* (9)	613 ± 139 455 ± 110	0/22	0/22	3/12	2/12	8/22
		Mananthavady, Wayanad	25	M (19) F (6)	579 ± 211 408 ± 89	0/25	0/25	0/9	0/9	5/25
	Total tested		289			0/289	0/289	4/44	2/44	57/272

### 3.2 Nipah virus seroprevalence in *Pteropus medius*

Seropositivity rates of 9% (8/88 *P. medius*), 24% (18/74 *P. medius*), and 28% (31/110 *P. medius*) were observed in February, July, and September, respectively, using ELISA (Figure 1C). All seropositive samples (*n* = 23) tested using the NiV neutralization assay were found to have neutralizing antibody titers ≥10, as given in Supplementary Table S1. All seronegative samples (*n* = 12) tested were negative for neutralizing antibodies. All the NiV RNA detected *P. medius* bats were found seropositive. The *Rousettus* sp. bats were found seronegative.

## 4 Discussion

In a survey of 289 *P. medius* bats in Kerala in February, July, and September 2023, NiV RNA was not detected in oral or rectal swabs from the sampled bats. However, in a subset of randomly selected bats across the sampling period, 9% (4/44) of the bats had detectable Nipah virus RNA in the spleen or kidney samples. An overall low NiV RNA positivity was observed in *P. medius* bats in the present study. Varied virus detection rates of 25% (May 2018), 2.75% (June 2019), and 0% (September 2021) have been observed in Kerala during previous outbreaks (Yadav et al., 2019, 2022; Sudeep et al., 2021). A longitudinal study conducted in Faridpur, Bangladesh, estimated a NiV prevalence of 0 to 3% in *P. medius* (Epstein et al., 2020). The NiV infection dynamics in *P. medius* are not fully understood and are thought to be cyclical and associated with the waning immunity and re-introduction of the virus to colonies through bat immigration or virus recrudescence (Epstein et al., 2020). Longitudinal or experimental studies in *P. medius* could offer more insights into the infection kinetics. The similarity in the viral genome with earlier outbreaks (2018, 2019, 2021) strains from the same region indicates a stable genotype circulation.

Field studies from India, and Bangladesh have shown NiV detection in oral, rectal, and urogenital swabs and urine, kidney, spleen, liver, heart, intestine, and reproductive organ samples of *P. medius* (Yadav et al., 2018, 2019; Epstein et al., 2020; Sudeep et al., 2021). Studies in Bangladesh have shown urine, urogenital swabs, and oral swabs showing higher virus detection than rectal swabs (Epstein et al., 2020). In 2010, the liver homogenate of a *P. medius* bat from Maynaguri, West Bengal, showed the presence of NiV by quantitative RT-PCR (Yadav et al., 2012). The liver, spleen, and kidney samples of the bats collected from Cooch Bihar district, West Bengal, and Dhubri district, Assam, showed NiV presence, whereas the throat and rectal swabs collected were negative for NiV (Yadav et al., 2018). In 2018, real-time RT-PCR analysis detected NiV RNA positivity in throat and rectal swabs of 25% (13/52) *P. medius* and organ samples (liver and spleen) of 5.7% (3/52) of bats collected from Kozhikode (Yadav et al., 2019). We have chosen the throat and rectal swab samples for the screening as per the observation from the above study. Here, we have mostly relied on the throat and rectal swab samples for testing, and the urine and urogenital swabs were not collected, which could also have limited the virus detection rate. This also indicates the absence of active viral shedding through the oral or gastrointestinal tract. The under-roost sampling for droppings/urine is less feasible around the year in Kerala due to the abundant rainfall.

We observed 9, 24, and 28% seropositivity in *P. medius* bats during the months of February, July, and September, respectively. A seroprevalence of 21% in *P. medius* was reported during outbreak investigations in Kerala during 2019 and 2021 (Sudeep et al., 2021; Yadav et al., 2022). Even a similar seroprevalence of approximately 20% was observed during non-outbreak sampling times, i.e., in and November 2019 from the state (Gokhale et al., 2022). Here, we have observed lower seropositivity in February compared to July and September, which could be due to the convenient sampling strategy too. Such fluctuating seroprevalence in bat populations has been described earlier in Bangladesh and Malaysia (Rahman et al., 2013; Epstein et al., 2020). The longitudinal study of NiV serodynamics in *P. medius* adults in Faridpur, Bangladesh, from 2006 to 2012 estimated a seroprevalence ranging from 30 to 80% (Epstein et al., 2020). In *Pteropus hypomelanus* and *Pteropus vampyrus* found in Malaysia, NiV seroprevalence ranging between 10 and 30% has been documented (Rahman et al., 2013).

*Pteropus medius* is widely distributed in Kerala, with colonies near human habitats (Raman et al., 2021). The foraging of fruit trees and orchards near human settlements could be a possible risk factor for humans. The virus positivity detected in the present study was from roosts located approximately 40–60 km far from the outbreak regions in Kozhikode. Even though this distance does not fall under the typical foraging ranges of 15–45 km of *P. medius*, roost shifts to more than 100 km distance have been documented for *P. medius* (Murugavel et al., 2023). A reproductive behavior study from South India has reported an increase in the *P. medius* colony size during the rainy season, which also coincides with the mating season from July to October (Jeevan et al., 2017). Few reports of mating behavior in other seasons, i.e., from February to June, are also documented (Rao, 2017). The polygynandrous mating system and increased contact during the breeding season can drive virus transmission and maintenance among bats. The present study has limited data to derive any relation between breeding behavior and NiV shedding. Longitudinal studies are necessary to understand reproductive behavior, bat population changes over time, and any possible correlation with virus transmission among bats.

The virus detection in bats coincided with the previous NiV outbreaks in the region, supporting the hypothesis of sporadic infection through spillover. However, epidemiological studies identifying consistent risk factors for NiV transmission from bats to humans are not available from India. Detailed case–control studies are needed to identify routes of spillover from bats to humans as well as longitudinal studies of Nipah virus shedding and serology in *P. medius* to understand the ecology of this virus in Indian bats. Virus detection in bats from the different locations studied cautions that the spillover risk exists in the region and necessary precautions should be taken.

## Data availability statement

The datasets presented in this study can be found in online repositories. The names of the repository/repositories and accession number(s) can be found in the article/Supplementary material.

## Ethics statement

The animal study was approved by Institutional Animal Ethics Committee, ICMR-National Institute of Virology, Pune. The study was conducted in accordance with the local legislation and institutional requirements.

## Author contributions

RB: Conceptualization, Investigation, Methodology, Supervision, Writing – review & editing. SM: Conceptualization, Formal analysis, Investigation, Methodology, Supervision, Writing – original draft, Writing – review & editing. UT: Investigation, Methodology, Supervision, Writing – review & editing. AS: Formal analysis, Investigation, Methodology, Supervision, Validation, Writing – review & editing. DiP: Investigation, Methodology, Writing – review & editing. KS: Investigation, Methodology, Writing – review & editing. BM: Investigation, Methodology, Writing – review & editing. RS: Methodology, Writing – review & editing. DeP: Methodology, Writing – review & editing. PY: Conceptualization, Data curation, Formal analysis, Funding acquisition, Project administration, Resources, Supervision, Visualization, Writing – review & editing.
