# Supplementary material for: Surveillance of Nipah virus in Pteropus medius of Kerala state, India, 2023
Source: Front Microbiol. 2024 Mar 5;15:1342170. doi: 10.3389/fmicb.2024.1342170 (PMC10951996; doi:10.3389/fmicb.2024.1342170)
Supplement: Supplementary file 1 [file Table_1.DOCX]

| **Month of sample collection** | **No.** | **ELISA results** | **MNT titer** |
| --- | --- | --- | --- |
| February, 2023 | 1 | Positive | 320 |
|  | 2 |  | 320 |
|  | 3 |  | 640 |
|  | 4 |  | 320 |
|  | 5 | Negative | 0 |
|  | 6 |  | 0 |
|  | 7 |  | 0 |
|  | 8 |  | 0 |
| July, 2023 | 1 | Positive | 20 |
|  | 2 |  | 80 |
|  | 3 |  | 20 |
|  | 4 |  | 320 |
|  | 5 |  | 20 |
|  | 6 |  | 20 |
|  | 7 |  | 80 |
|  | 8 |  | 10 |
|  | 9 | Negative | 0 |
|  | 10 |  | 0 |
|  | 11 |  | 0 |
|  | 12 |  | 0 |
| September, 2023 | 1 | Positive | 80 |
|  | 2 |  | 80 |
|  | 3 |  | 160 |
|  | 4 |  | 10 |
|  | 5 |  | 20 |
|  | 6 |  | 40 |
|  | 7 |  | 20 |
|  | 8 |  | 80 |
|  | 9 |  | 10 |
|  | 10 |  | 640 |
|  | 11 |  | 320 |
|  | 12 | Negative | 0 |
|  | 13 |  | 0 |
|  | 14 |  | 0 |
|  | 15 |  | 0 |

**Supplementary Table 1:** The neutralization titer of the serum samples tested by microneutralization assay.
